# Supplementary material for: MetaRibo-Seq measures translation in microbiomes
Source: Nat Commun. 2020 Jun 29;11:3268. doi: 10.1038/s41467-020-17081-z (PMC7324362; doi:10.1038/s41467-020-17081-z)
Supplement: Supplementary file 10 — Supplementary Data 7 [file 41467_2020_17081_MOESM10_ESM.zip › File2/Confidence_High_Taxonomy/79335_out.krona.html]

Javascript must be enabled to view this page.

members
magnitude
magnitudeUnassigned
count
unassigned
taxon
rank

79335\_out

4

superkingdom
2
4

1239
4
phylum

class
4
186801

4
186802
order

541000
4
family

species

SRS051610\_contig\_number\_62SRS053214\_contig\_number\_contig-100\_0.1SRS148196\_contig\_number\_contig-100\_2.2SRS148424\_contig\_number\_contig-100\_1200.1200
4
1898205
